# GLP-1 receptor agonist for weight loss and fertility: Social media and online perception *versus* evidence-based medicine

**DOI:** 10.1371/journal.pone.0326210

**Published:** 2025-07-02

**Authors:** Zaher Merhi, Manasi Karekar, Marco Mouanness

**Affiliations:** 1 Department of Obstetrics and Gynecology, Division of Reproductive Endocrinology and Infertility, Maimonides Medical Center, Brooklyn, New York, United States of America; 2 Department of Biochemistry, Albert Einstein College of Medicine, Bronx, NY, United States of America; 3 Reproductive Endocrinology and Infertility, Rejuvenating Fertility Center, New York, New York, United States of America; The Chinese University of Hong Kong, HONG KONG

## Abstract

**Background:**

The injectable glucagon-like peptide-1 (GLP-1) receptor agonist (RA) drugs are commonly used for weight loss among reproductive-aged women but the perception of their impact on fertility is not clear among the public.

**Objectives:**

The purpose of this review was to compare scientific literature to social media discourse and online search pertaining to the potential impact of GLP-1 RAs on female fertility.

**Methods:**

Group 1 included 3 social media platforms, each of which required a different data collection method. For Reddit, VADER (Communalytic ®) was used as a sentiment analyzer and for Twitter and TikTok, we performed a manual search for posts and scored them using an objective internal scale. Group 2 included the online search engine Google Trends. Group 3 consisted of medical literature search by PubMed. All sentiments of posts/comments/articles were graded as: “Positive, Neutral, or Negative” and compared among groups.

**Results:**

In Groups 1 and 2, scores showed a significantly more Positive than Neutral, and more Neutral than Negative sentiments. In Group 3, among the 52 original studies found on PubMed in women with polycystic ovary syndrome (PCOS), they all had Positive sentiments. In Groups 1, 2, and 3, there was a strong positive correlation among all sentiments (r^2^ = 0.83). Even though all 3 social media platforms had the majority of posts with a Positive sentiment and correlated with Google Trends posts and with PubMed studies in women with PCOS (r^2 ^= 0.74), there was a lack of PubMed studies pertaining to the effect of GLP-1 RA in women without PCOS.

**Conclusion:**

The Positive sentiments among women without PCOS is not justified by evidence-based medicine and there is a clear need for studies pertaining to this topic.

## Introduction

Glucagon-like peptide-1 (GLP-1) is a natural hormone that is released by the gastrointestinal system and plays an important tole in glucose homeostasis [1]. It acts on GLP-1 receptors that are abundant in the pancreas leading to increased insulin secretion and inhibition of glucagon secretion thus lowering blood glucose levels [1]. The endogenous GLP-1 also lowers gastric emptying and increases food satiety. It slows gastric emptying, thus delaying the passage of food from the stomach into the small intestine. This prolongs the feeling of fullness after meals, leading to reduced food intake. By increasing satiety and lowering the rate at which food is digested and absorbed, GLP-1 contributes to weight loss and improved glycemic control. GLP-1 receptor agonists (GLP-1 RAs) are medications that have been used in the treatment of diabetes and its comorbidities such as cardiovascular disease [2] and renal disease [3]. Additionally, GLP-1 RAs have been shown in randomized trials to cause a sustained weight loss [4,5] most likely by acting both centrally at the level of the brain and at the peripheral tissues [6].

Since its FDA approval for weight loss, the use of GLP-1 RAs for weight loss has rapidly gained popularity on social media, especially among obese women of reproductive-age [7]. The use of these antidiabetic medications is now easily available for purchase online and the trends on social media has caused an rapid surge in both demand and purchase of these weight loss drugs [8]. Since GLP-1 receptors exist in the reproductive organs [9], empirical results indicate that they could impact the reproductive system including the hypothalamic-pituitary-ovarian (HPO) axis. The impact of these medications on female fertility has been studied in women with polycystic ovary syndrome (PCOS) [10] where the results have shown that GLP-1 RAs could help women with PCOS both metabolically and reproductively [11,12]. For instance, a meta-analysis study of randomized trials compared metformin to GLP-1 RAs in women with PCOS and reported that GLP-1 RAs, in addition to lowering body weight, was significantly better than metformin in improving insulin sensitivity as well as improving menstrual cyclicity, lowering serum total testosterone, lowering total cholesterol, and lowering blood pressure [11].

As for the impact of GLP-1 RAs on fertility status in women without PCOS, at the time of writing this manuscript, a detailed literature search resulted in finding a few studies in non-PCOS female animal model but none in humans [9,13–15]. The aim of this study was to compare women’s sentiment between social media perception and online search *versus* evidence-based medicine toward the GLP-1 RAs and their impact on fertility.

## Materials and methods

### Data collection

Data were collected from three main sources: from social media platfoms that included Reddit, Twitter, and TikTok (Group 1), from the large online search Google Trends (Group 2), and from Pubmed which is a free and publicly available resource provided by the US National Library of Medicine (Group 3). These three social media platforms and the Google Trends were chosen because they have a global reach, a high number of users, and significant influence on online and social media interactions and content sharing. Only publicly available data from the selected social media platforms were analysed and there was no access to any individual accounts. The same keywords pertaining to GLP-1 RAs and fertility were used for data collection for all 3 groups (Table 1). Because the data was public, informed consent from participants was not needed and the study was IRB exempt. For all data collected, comments or studies that are not in English language. Addionally trivial or not relevant comments to the subject were excluded.

**Table 1:** List of all the GLP-1 RA and fertility-related keywords used for data collection in the study.

GLP-1 RA related keywords	Fertility-related keywords
Ozempic	infertility
Dulaglutide	in vitro fertilization
Trulicity	IVF
Exenatide	reproductive
Liraglutide	reproductive health
Victoza	conception
Semaglutide	ovulation
Byetta	reproductive endocrinology
Bydureon	fertility specialist
Lixisenatide	fertility clinic
Adlyxin	polycystic ovary syndrome
GLP-1	PCOS
GLP1	ovarian cysts
GLP-1 RA	hormone imbalance
GLP1 RA	menstrual irregularities
GLP-1 receptor agonist	menstruation
GLP1 receptor agonist	menses
weight-loss medication	period
weight loss drug	ovarian reserve
weight-loss injection	ovulatory disorders
obesity treatment	endometriosis
obesity medication	female infertility
Obesity	maternal health
Obese	embryo
overweight	embryo transfer
	embryo freezing
	egg freezing
	fertility preservation
	oocyte physiology
	granulosa cell

### Data extraction in Group 1

#### a. *From the social platform Reddit.*

Reddit is a web-based platform that organizes topics into subreddits (distinct forums) where each interaction/discussion is considered a thread [16]. Reddit is very popular and its users, called Redditors, discuss a variety of topics including infertility treatments and weight loss using the new injectable medications such as Ozempic and others [17]. Reddit posts, which are anonymous and voluntary, have become a common source for discussing research studies and important publications worldwide [16].

The computational social science research tool Communalytic https://edu.communalytic.org/ was used to extract data from online communities and discourse on the social platform Reddit. VADER (Valence Aware Dictionary and Sentiment Reasoner), a pre-trained sentiment analysis model that provides a sentiment score for a given text, was used to score the sentiment of the latest 200 posts as: Positive, Negative, or Neutral. Within the threads, the posts were filtered including keywords related to fertility using an advanced search query that included the keywords in Table 1. Using Communalytic’s sorting filters, the top 200 most recent submissions were selected, including replies and comments, while excluded reposts and duplicates. Finally, we ran Communalytic on four subreddits that were analyzed with and without filter for each subreddit: r/Infertility, r/IVF, r/Ozempic, and r/Semaglutide, leading to 8 datasets.

#### b. *From the social media platforms Tiktok and Twitter.*

Unlike Reddit,Twitter and TikTok do not allow a third party software analysis so we manually searched the latest 200 posts on these platforms using the same keywords in Table 1 to assess whether they mention Positive, Negative, or Neutral sentiment using an objective internal scale. The internal scale included the 3 following criteria within the search process: use of evidence, language/tone, and engagement/response (Table 2).

**Table 2:** Ojective internal scale using three criteria for assessing sentiment on Twitter and TikTok.

	Positive sentiment	Negative sentiment	Neutral sentiment
**Use of Evidence**	Use of personal anecdotes, success stories, or scientific research supporting the benefits of GLP1 drugs for fertility	Use of personal anecdotes, concerns about side effects, or conflicting research indicating potential harm	Presentation of information without anecdotal evidence or biased research
**Language/tone**	Framing GLP1 drugs as beneficial tools in the infertility journey, using language that suggests optimism and hope	Framing GLP1 drugs as risky or potentially harmful, using language that suggests caution or skepticism	Presentation of information without strong emotional language or biased framing
**Engagement/response**	Comments expressing agreement, support, or gratitude towards the content	Comments expressing disagreement, concern, or criticism towards the content	Mix of comments or minimal engagement without strong emotional reactions

### Data extraction from Google Trends (Group 2)

Google Trends provides keyword-related data including search volume index and geographical information about search engine users. It can be used for comparative keyword research and to discover event-triggered spikes in keyword search volume. Google Trends also allows the user to compare the relative search volume of searches between two or more terms. The values in Google Trends ranges from 0 to 100, representing search interest in different regions and times. A value of 0 indicates that the search queries are not popular enough for this search term. A value of 50 indicates that the search term is half as popular. A value of 100 indicates that the search term has peak popularity. In this study, we defined the region as “United States”, and category as “Health” on the Google Trends website. A total of 126 million results using the combination of keywords in Table 1 were found.

### Data extraction from PubMed (Group 3)

A Pubmed search was done using the keywords in Table 1. We performed a detailed systematic review of all *in vitro* and *in vivo* studies, all prospective and retrospective studies, as well as basic science and clinical studies that are available in Pubmed in English language. The references from all relevant articles were checked and we performed a search of all abstracts of the annual meetings of the American Society for Reproductive Medicine (ASRM) and the European Society for Human Reproduction and Embryology (ESHRE). We reviewed all the titles and abstracts of all citations. The data were extracted from the text, and all the tables and graphs within the manuscripts. The reference lists of identified articles were searched for additional references. All data were abstracted and put into a table format in a systematic manner. Exclusion criteria included editorials, letters to editors, and studies pertaining to male subjects.

### Statistics

Data are expressed as mean ± standard error of the mean. A sample size of 42 posts on TikTok or Twitter has been shown to be adequate to produce statistical difference with 80% power and two-tailed α error of 0.05. Because there were 3 groups, ANOVA with post-hoc analysis was used to determine which specific groups were statistically different. For categorial data, Chi-square test was used to compare sentiments. To assess whether there was a positive or negative association, Pearson linear regression was performed on data extracted from all three groups. R^2^ was calculated to assess the strength of the correlations and p < 0.05 was considered statistically significant. Prism 10 software was used for all statistical analyses.

## Results

### Group 1- Reddit social platform subgroup

In Group 1, on Reddit social platform, out of 46,972 total number of records analyzed including all post headers, comments, replies and excluding reposts and duplicates, there was a significantly higher Positive sentiment compared to Neutral sentiment (4,720 ± 1,669 *vs.* 659 ± 243; respectively, p = 0.027; Table 3). There was trend towards higher score for Positive sentiment compared to Negative sentiment (p = 0.068). Negative and Neutral sentiments has similar scores (p = 0.88).

**Table 3:** Comparison between sentiments on the Reddit platform.

	Difference	Range	p-value
**Neutral sentiment** vs. **Negative sentiment**	−661.1	−4280–2958	0.88
**Positive sentiment** vs. **Negative sentiment**	3399	−219.6 to 7018	0.068
**Positive sentiment** vs. **Neutral sentiment**	4061	441.5 to 7680	0.027

When looking into the data in more details, the most common topics were “IVF, Infertility, Semaglutide, and Ozempic.” Thus, we performed Communalytic on these 4 topics, with and without filter for each topic leading to 8 datasets (Table 4). In the majority of scores, Positive sentiment had significantly higher score than Negative sentiment that had significantly higher score than Neutral sentiment (p < 0.0001) except in the sematuglide with filters where the Negative and Neutral sentiments were similar (p = 0.22).

**Table 4:** Communalytic on 8 datasets that included four topics; with and without filters for each topic.

Reddit thread name (r/____)	Total # of records	Positive sentiment	Neutral sentiment	Negative sentiment	p-value
**IVF**	13530	9631 (71.49%) ^a^	970 (7.20%) ^b^	2870 (21.31%) ^c^	<0.0001 (a *vs.* b *vs.* c)
**IVF + filters**	3754	2411 (64.41%) ^a^	322 (8.60%) ^b^	1010 (26.98%) ^c^	<0.0001(a *vs.* b *vs.* c)
**Infertility**	13392	8635 (64.48%) ^a^	1092 (8.15%) ^b^	3665 (27.37%) ^c^	<0.0001 (a *vs.* b *vs.* c)
**Infertility + filters**	3646	2457 (67.39%) ^a^	272 (7.46%) ^b^	917 (25.15%) ^c^	<0.0001 (a *vs.* b *vs.* c)
**Semaglutide**	13882	9996 (72.01%) ^a^	1613 (11.62%) ^b^	2273 (16.37%) ^c^	<0.0001 (a *vs.* b *vs.* c)
**Semaglutide + filters**	171	102 (60.00%) ^a^	30 (17.65%) ^b*^	38 (22.35%) ^b*^	<0.0001 (a *vs.* b)
**Ozempic**	11636	8272 (71.09%) ^a^	1346 (11.57%) ^b^	2018 (17.34%) ^c^	<0.0001 (a *vs.* b *vs.* c)
**Ozempic + filters**	353	171 (48.58%) ^a^	63 (17.90%) ^b^	118 (33.52%) ^c^	<0.0001(a *vs.* b *vs.* c)

*p= 0.22.

### Group 1- Tiktok and Twitter platforms subgroup

In Group 1, on TikTok, out of 70 posts, Positive sentiment had significantly higher score than Negative or Neutral sentiments (p = 0.006; Table 5) while Negative and Neutral sentiments were similar (p = 0.06). On Twitter, out of 88 posts, Positive sentiment had significantly higher score than Neutral sentiment that had significantly higher score than Negative sentiment (p = 0.001; Table 5).

**Table 5:** Results of the data extracted from Tiktok and Twitter platforms.

	Tiktok (out of 200 total posts)	Twitter (out of 200 total posts)
**Number of posts related to fertility**	70	88
**Positive sentiment**	37 (51.85%) ^a^	48 (54.54%) ^a^
**Neutral sentiment**	21 (30%) ^b^	29 (32.95%) ^b^
**Negative sentiment**	12 (17.14%) ^b^	11 (12.5%) ^c^
**p-value**	0.006 (a *vs.* b)	0.001 (a *vs.* b *vs.* c)

### Group 2- Google Trends

In Group 2, there was a peak spike in interest in Ozempic and fertility treatments with the top 5 states involved being Massachusetts, California, New York, Texas, and Florida. The terms “Ozempic fertility”, “Ozempic Babies” and “Ozempic getting pregnant” had significant increase in the last year reaching +600%, + 1100%, and 900% respectively. When the term “Ozempic” or “GLP-1 RA” were searched, there was a + 800% increase in the search for the term “fertility” and +650% increase in the search for the term “pregnancy” as a follow up search.

Overall search showed that Positive sentiment had significantly higher score than Neutral sentiment that had significantly higher score than Negative sentiment (p < 0.0001). When the data were evaluated in more details, we found that the rank for top 5 search terms was as follows: Ozempic fertility, Ozempic pregnancy, Ozempic weight loss, Ozempic PCOS, and Ozempic babies. Table 6 shows the sentiment analysis of the most common 200 posts on Google Trends related to the two most common topics “Ozempic Fertility” and “Ozempic Pregnancy.” For “Ozempic Fertility,” the majority had a Positive sentiment which had significantly higher score than Neutral sentiment that had significantly higher score than Negative sentiment (p = 0.03; Table 6); however for “Ozempic Pregnancy,” the majority had a Neutral sentiment that had significantly higher score than Positive sentiment that had significantly higher score than Negative sentiment (p < 0.0001; Table 6).

**Table 6:** Sentiment analysis of the first 200 posts on Google related to two main topics.

	Positive sentiment	Neutral sentiment	Negative sentiment	p-value
**“Ozempic Fertility”**	97 (48.5%) ^a^	61 (30.5%) ^b^	42 (21%) ^c^	0.03 (a *vs.* b *vs.* c)
**“Ozempic Pregnancy”**	48 (24%) ^a^	134 (67%) ^b^	18 (9%) ^c^	<0.0001 (a *vs.* b *vs.* c)

### Comprison between Group 1 and Group 2

Group 1 subgroups and Group 2 has similar percentage of Positive (p = 0.9), Neutral (p = 0.8), and Negative (p = 0.7) sentiments. Among each type of sentiment, there was a strong positive correlation among all Group 1 subgroups and Group 2 (r^2^ = 0.83). Positive sentiments had significantly higher score than Neutral sentiment that had significantly higher score than Negative sentiment in all (Table 7).

**Table 7:** Comparison between the sentiments within the social media platforms and Google Trends.

	Positive sentiment	Neutral sentiment	Negative sentiment	p-value
Reddit (n = 46,972)	54.00% ^a^	23.30% ^b^	22.70% ^c^	0.02 (a *vs.* b *vs.* c)
TikTok (n = 70)	51.85% ^a^	31.01% ^b^	17.14% ^c^	0.03 (a *vs.* b *vs.* c)
Twitter (n = 88)	54.54% ^a^	32.95% ^b^	12.51% ^c^	0.03 (a *vs.* b *vs.* c)
Google Trends (n = 200)	48.50% ^a^	30.50% ^b^	21.00% ^c^	0.03 (a *vs.* b *vs.* c)
**p-value**	0.9	0.8	0.7	

### Group 3- PubMed

Results are shown in Table 8. The majority of peer-reviewed manuscripts were in PCOS state both in animal and humans. In women without PCOS, there were no studies (at the time of the publication). Although majority of the posts in all three social media platforms showed positive sentiment, there are no studies in women without PCOS (n = 0) that would justify that positivity among public perception. Among the 52 original studies on PubMed in women with PCOS, they all had positive sentiments. There were no studies in humans (including case reports, case series, cohort, case-control, or trials) evaluating the impact of GLP-1 RAs on ovulation, implantation, IVF, or any fertility status in women without PCOS, but there were a few studies in animals that showed controversial results with negative impact on fertility.

**Table 8:** Peer reviewed studies pertaining to GLP-1 RA and infertility related keywords.

Pubmed search for “GLP-1 agonist with”	Peer-reviewed studies
**PCOS**	112 total:
	52 original articles
	60 review article
**Without PCOS**	0
**Ovarian reserve**	1 in animal model
**Oocyte physiology**	1 in animal model
**Menstrual irregularities**	1 review article
**IVF**	1 original article in PCOS
**Granulosa cell physiology**	3 in animal model
**Endometriosis**	0

## Discussion

In this study, we assessed the sentiments of women of reproductive age pertaining to GLP-1 RA injectable weight loss injectables and their impact on fertility. In a comparison between three major social media platforms (Twitter, Reddit, TikTok), the largest online search engine (Google), and a large scientific medical library (PubMed), we found most content to have a positive sentiment. There was a positive correlation between the sentiment of social media content and evidence-based medicine; however, evidence-based medicine justified this positivity only in women with PCOS.

Studies using social media data face several limitations. Self-reported information can be inaccurate or incomplete, and the users providing these reports may not reflect the general population’s social, economic, or demographic diversity. The presence of fake accounts may also impact data quality. These limitations highlight the need for cautious interpretation and support the integration of other research methods. Additional concerns include researcher bias, language variability, and users’ self-presentation, which may influence data reliability. The exclusive use of English and specific keywords could introduce selection bias and exclude relevant content. Differences in user demographics across platforms further limit generalizability. Additionally, this study did not compare specific GLP-1 RAs that are used at different frequency (for example: tirzepatide *versus* semaglutide) or administration methods of these drugs.

Even though we have used objective measures with scores that allowed for systematic classification to assess sentiments such as VADER, a pre-trained sentiment analysis model that provides a sentiment score for a given text, and Google Trends, that gives values from 0 to 100, they also carry limitations. For example, sarcasm, slang, or context-dependent expressions may be misclassified by automated tools, leading to potential distortions in sentiment interpretation. Furthermore, certain health-related keywords (e.g., “pain,” “weight,” or “injection”) might inherently carry a negative tone, even in neutral or informative contexts. Such factors could influence the overall sentiment trends observed in the data, and may over- or under-estimate public perception. Different platforms (e.g., Twitter, Instagram, Reddit) have varying norms for language, tone, and engagement. What’s considered “neutral” on Reddit might be “negative” on Instagram. Finally, reducing sentiment to only three categories (positive, negative, neutral) may overlook mixed sentiments, sarcasm, or nuanced discussions that include both support and concern. Therefore, while sentiment scores provide a valuable quantitative overview, findings should be interpreted with caution and ideally validated through manual review or triangulated with qualitative methods.

### Do GLP-1 RAs improve reproduction in women with PCOS?

The GLP-1 receptors exist in the hypothalamus, pituitary, and reproductive organs like the ovaries and the uterus [9]. Thus it is logical to expect an impact of the new injectable weight loss drug, GLP-1 RAs, on female reproduction [18,19]. It is well established that a healthy lifestyle and weight loss-inclusive care in obese women with PCOS could lead to an improvement in both metabolic and reproductive disturbances which could ultimately lead to restoring anovulation and improving fertility [20]. Metformin has been used as a gold standard therapy for women with PCOS who have glucose intolerance, regardless of weight [20].

The recent GLP-1 RAs have been relatively well-studied in obese women with PCOS; the data in both humans and animals, pertaining to weight loss and visceral fat tissue, insulin resistance, menstrual irregularities, gut microbiota, fatty liver disease, and even cardiometabolic parameters in PCOS, are reassuring thus far [21–32]. A meta-analysis of randomized trials (n = 8) compared metformin alone to GLP-1 RA injectables in women with PCOS and showed that that GLP-1 RAs significantly improved insulin resistance, blood pressure, cholesterol and menstrual irregularities as well as lowered weight/abdominal circumference along with androgen indices and hirsutism, in a manner better than metformin alone [11].

In the preconception period, the intake of metformin with (Group 1) or without (Group 2) liraglutide, a GLP-1 RA, for 3 months was studied in a randomized open-label study on IVF outcomes in obese PCOS women [33]. Even though both Groups 1 and 2 had a similar weight loss amount, Group 1 had a significantly higher pregnancy rate after IVF (i.e., following embryo transfer) compared to Group 2 (85.7% *versus* 28.6%, respectively; p = 0.03). Their findings indicated that it was not the amount of weight loss alone that contributed to a better outcome in Group 1 (since both groups had a similar weight loss amount) and that GLP-1 RAs might be acting directly and favorably on the reproductive system in these women. To further explore the effect of GLP-1 RAs on the ovaries at the cellular level, a study evaluated their impact on granulosa cells from a PCOS mouse model [34] and showed a favorable effect on granulosa cell proliferation (and reducing apoptosis) via forkhead box protein O1 (FoxO1) [34].

Taking these findings into consideration, GLP-1 RAs seem to improve reproductive potential in PCOS state via two mechanisms: indirectly via weight loss and directly by acting on the ovaries [35]. These findings are reassuring because they do correlate with our findings extracted from the public via three social media platforms and Google search.

### Any justification for the use of GLP-1 RAs in women without PCOS?

At the time of writing this manuscript and after performing an extensive literature search on PubMed, there were no studies in humans evaluating the impact of GLP-1 RAs on fertility status in women without PCOS, but there were a few studies in animals that showed controversial results with negative impact on fertility.

A study investigated the impact of subcutaneous GLP-1 RA liraglutide administration over 5 weeks on ovarian and uterine tissues in rats [13]. The results showed unfavorable alterations in hormone levels; for instance, there was a significant increase in androgens and a significant decrease in serum LH, follicle-stimulating hormone (FSH), estradiol (E2), and progesterone (P4) that were associated with ovarian weight loss [13]. Another unfavorable result included a significant drop in ovarian antioxidant molecules such as glutathione, catalase, and superoxide dismutase levels as well as toxic effects of liraglutide on granulosa cells and ovarian follicles (fewer funcational follicles, more atretic follicles, more apoptosis, and more fibrous tissue) [13]. Similar unfavorable results were seen at the uterine level as reflected by destruction of the epithelium and endometrial stroma with more apoptosis [13]. Another study demonstrated that GLP-1, in its natural form, caused a significant suppression of FSH-induced P4 synthesis and FSH-induced cAMP production in rat granulosa cells as well as downregulation of the steroidogenic enzymes StAR, P450scc and 3β-HSD [9]. Another study showed that the GLP-1 RA Exendin-4 (Ex4) also had an adverse impact on the HPO axis [14] by causing a significant drop in LH levels leading to LH surge suppression via reducing the *Kiss-1* and *Kiss-1r* expression in the hypothalamus and a delay in puberty, as reflected by vaginal opening [14].

Even though the studies were performed on animals only and did not show benefit from GLP-1 RAs on reproduction, they cannot be translated to humans. The findings so far could be worrying since the positive sentiment on social media and online searches is not supported by evidence-based medicine in women without PCOS.

## Conclusion

Women with PCOS are a unique population that is very distinct than than those without PCOS because they could have metabolic dysfunction that understandably would benefit from the anti-diabetic actions of the GLP-1 RAs. The vulnerability of this infertility population and the wealth of online and social media misinformation (such as “#ozempicbabies” and “#ozempicpregnancy”) is causing a dramatic rise in the use of the quick fix GLP-1 RA medications with their unknown reproductive consequences [36]. There is a clear need for long-term studies in women without PCOS, to assess the impact of GLP-1 RA medications in a dose-response manner on reproduction to evaluate their safety since many women of reproductive age are resorting to these medications without knowing whether they could have any negative impact on their future fertility.

## Supporting information

S1 Data RAW DATA- GLP1 and google. (DOCX)

S2 Data RAW DATA- GLP-1 drugs and social media. (DOCX)
