# Supplementary material for: GLP-1 receptor agonist for weight loss and fertility: Social media and online perception versus evidence-based medicine
Source: PLoS One. 2025 Jul 2;20(7):e0326210. doi: 10.1371/journal.pone.0326210 (PMC12221084; doi:10.1371/journal.pone.0326210)
Supplement: S1 Data — (DOCX) [file pone.0326210.s001.docx]

| GLP-1 | obesity |
| --- | --- |
| , GLP-1 receptor agonist | , weight loss |
| , Ozempic | , fertility |
| , Dulaglutide | , infertility |
| , Trulicity | , pregnant |
| , Exenatide | , pregnancy |
| , Byetta, | , PCOS |
| Bydureon | , IVF |
| , Liraglutide | , period |
| , Victoza | , menstruation |
| , Lixisenatide | , embryo |
| , Adlyxin | , ovulation |
| , Semaglutide | , ovarian reserve |
|  | , reproductive |

LIMTED THE SEARCH PERIOD TO 01/01/2024-04/20/2024:

None of the combinations had a pubmed link in the first 10 suggested results.

Total 126 million results using the combination of these words during the period of 1/1/24-4/20/24

**Google Trends :**

Google Trends provides keyword-related data including search volume index and geographical information about search engine users. It can be used for comparative keyword research and to discover event-triggered spikes in keyword search volume. Google Trends also allows the user to compare the relative search volume of searches between two or more terms. The values in Google Trends ranges from 0 to 100, representing search interest in different regions and times. A value of 0 indicates that the search queries are not popular enough for this search term. A value of 50 indicates that the search term is half as popular. A value of 100 indicates that the search term has peak popularity. In this study, we defined the region as “United States”, category as “Health” and custom time range as “1/1/2024–4/20/2024” on the Google Trends website.

Results showed:

- A spike in interest in Ozempic and fertility treatments in March and April of 2024 (with the peak being on March 30 2024), with the top 5 states involved being Massachusetts, California, New York, Texas and Florida.
- The terms “Ozempic fertility”, “Ozempic Babies” and “Ozempic getting pregnant” had significant increases during that period, reaching +600%, +1100% and 900% respectively.
- When the term “Ozempic” or “GLP1-agonist” was searched, there was a 800% increase in the search for the term “fertility” and 650% increase in the search for the term “pregnancy” as a follow up search
- In the past 90 days (period of Jan 20 to April 20 2024), the rank for top 5 search terms was as follows:

Ozempic weight loss, Ozempic PCOS, Ozempic fertility, Ozempic babies, Ozempic pregnant

- Sentiment Analysis of the first 200 posts on google related to “Ozempic Fertility” (omitted the sponsored content), limited to the period of 01/01/2024 to 4/20/2024:
- Positive: 97 (48.5%)
- Neutral: 61 (30.5%)
- Negative: 42 (21%)
- Sentiment Analysis of the first 200 posts on google related to “Ozempic Pregnancy” (omitted the sponsored content), limited to the period of 01/01/2024 to 4/20/2024:
- Positive: 48 (24%)
- Neutral: 18 (9%)
- Negative: 134 (67%), stating safety concerns mainly and recommendations to stop it prior to attempting pregnancy
