# Supplementary material for: GLP-1 receptor agonist for weight loss and fertility: Social media and online perception versus evidence-based medicine
Source: PLoS One. 2025 Jul 2;20(7):e0326210. doi: 10.1371/journal.pone.0326210 (PMC12221084; doi:10.1371/journal.pone.0326210)
Supplement: S2 Data — (DOCX) [file pone.0326210.s002.docx]

**Keywords for search**

| **GLP-1 related words** | **Infertility related words** |
| --- | --- |
| Dulaglutide  Trulicity  Exenatide  Liraglutide  Victoza  Semaglutide  Ozempic  GLP-1  GLP1  GLP-1 RA  GLP1 RA  GLP-1 receptor agonist  GLP1 receptor agonist  weight-loss medication  weight loss drug  obesity treatment  obesity medication  diabetes medication  diabetes drug  diabetes treatment  diabetes  obesity | in vitro fertilization  IVF  infertility clinic  reproductive health  conception  ovulation  reproductive endocrinology  fertility specialist  fertility clinic  polycystic ovary syndrome  PCOS  ovarian cysts  hormone imbalance  menstrual irregularities  ovarian reserve  ovulatory disorders  endometriosis  female infertility  maternal health  embryo transfer  embryo freezing  egg freezing  fertility preservation |

("Dulaglutide" OR "Trulicity" OR "Exenatide" OR "Liraglutide" OR "Victoza" OR "Semaglutide" OR "Ozempic” OR "GLP-1" OR "GLP1" OR "GLP-1 RA" OR "GLP1 RA" OR "GLP-1 receptor agonist" OR "GLP1 receptor agonist" OR "weight-loss medication" OR "weight loss drug" OR "obesity treatment" OR "obesity medication" OR "diabetes medication" OR "diabetes drug" OR "diabetes treatment" OR “diabetes” OR “obesity”) AND ("fertility" OR "infertility" OR "fertility treatment" OR "assisted reproductive technology" OR "in vitro fertilization" OR “IVF” OR "infertility clinic" OR "reproductive health" OR "conception" OR "ovulation" OR "reproductive endocrinology" OR "fertility specialist" OR "fertility clinic" OR "polycystic ovary syndrome" OR “PCOS” OR "ovarian cysts" OR "hormone imbalance" OR "menstrual irregularities" OR "ovarian reserve" OR "ovulatory disorders" OR "endometriosis" OR "female infertility" OR "maternal health" OR "embryo transfer" OR "embryo freezing" OR "egg freezing" OR "fertility preservation")

**Reddit**

“GLP-1 Drugs” vs “GLP-1 Drugs and Fertility” and “Infertility” vs “Infertility and GLP-1 drugs”

Methods for <https://edu.communalytic.org/>

- Searched reddit for threads related to GLP1 drugs and infertility
  - r/Ozempic and r/Semaglutide are the most popular ones; discussions probably include other GLP1 drugs too
  - r/IVF and r/Infertility
- Within these threads, I filtered the posts including keywords related to fertility using advanced search query
  - “fertility OR pregnant OR pregnancy OR pcos OR ivf OR period OR menstruation OR embryo OR ovulation OR ovarian reserve OR reproductive OR polycystic ovary syndrome OR ovarian cyst OR infertility”
  - "GLP-1" OR "GLP1" OR "Dulaglutide" OR "Trulicity" OR "Exenatide" OR "Byetta" OR "Bydureon" OR "Liraglutide" OR "Victoza" OR "Lixisenatide" OR "Adlyxin" OR "Semaglutide" OR "Ozempic" OR "weight loss medication" OR "weight loss drug" OR "diabetes" OR "obesity" OR "weight loss"
  - Sort submissions by “top”
  - 200 most recent submissions
  - “Yes” to collecting replies and comments
- Ran communalytic on the 8 datasets (four topics with and without filter)
  - Total number of posts includes main post + comments
  - Sentiment analysis: VADER English
  - Common words: used word cloud to see largest words

**Findings:**

| **Reddit thread name (r/____)** | **Total # of records** | **Negative sentiment** | **Neutral Sentiment** | **Positive Sentiment** |
| --- | --- | --- | --- | --- |
| **IVF** | 13530 | 2870 (21.31%) | 970 (7.20%) | 9631 (71.49%) |
| **IVF + filters** | 3754 | 1010 (26.98%) | 322 (8.60%) | 2411 (64.41%) |
| **Infertility** | 13392 | 3665 (27.37%) | 1092 (8.15%) | 8635 (64.48%) |
| **Infertility**  **+ filters** | 3646 | 917 (25.15%) | 272 (7.46%) | 2457 (67.39%) |
| **Semaglutide** | 13882 | 2273 (16.37%) | 1613 (11.62%) | 9996 (72.01%) |
| **Semaglutide**  **+ filters** | 171 | 38 (22.35%) | 30 (17.65%) | 102 (60.00%) |
| **Ozempic** | 11636 | 2018 (17.34%) | 1346 (11.57%) | 8272 (71.09%) |
| **Ozempic + filters** | 353 | 118 (33.52%) | 63 (17.90%) | 171 (48.58%) |

Total # of records: includes all post headers + comments + replies, excludes reposts/duplicates

| **Negative sentiment** vs. **Neutral sentiment** | 661.1 | -2958 to 4280 | No | ns | 0.8878 |
| --- | --- | --- | --- | --- | --- |
| **Negative sentiment** vs. **Positive sentiment** | -3399 | -7018 to 219.6 | No | ns | 0.0677 |
| **Neutral sentiment** vs. **Positive sentiment** | -4061 | -7680 to -441.5 | Yes | * | 0.0266 |

**TikTok & Instagram**

Criteria for assessing sentiment

- Use of Evidence:
  - Positive: Use of personal anecdotes, success stories, or scientific research supporting the benefits of GLP1 drugs for fertility
  - Negative: Use of personal anecdotes, concerns about side effects, or conflicting research indicating potential harm
  - Neutral: Presentation of information without anecdotal evidence or biased research
- Language/tone:
  - Positive: Framing GLP1 drugs as beneficial tools in the infertility journey, using language that suggests optimism and hope
  - Negative: Framing GLP1 drugs as risky or potentially harmful, using language that suggests caution or skepticism
  - Neutral: Presentation of information without strong emotional language or biased framing.
- Engagement/response:
  - Positive: Comments expressing agreement, support, or gratitude towards the content
  - Negative: Comments expressing disagreement, concern, or criticism towards the content
  - Neutral: Mix of comments or minimal engagement without strong emotional reactions.

Methods: using the following search terms, I went through 200 posts and assessed whether:

1. They mention anything about reproductive health (IVF, PCOS, infertility, pregnancy, etc)
2. It’s a positive, negative or neutral piece of content, using above criteria
3. Made notes about the content for more detail about why something was negative/neutral/positive.

|  | **Tiktok** | **Twitter** | **Instagram** |
| --- | --- | --- | --- |
| **Total posts** | 200 | | |
| **Search words** | - ozempic  - ozempic and fertility  - ozempic PCOS  - GLP1  - GLP1 pregnancy  - ozempic pregnancy | | - Ozempic  - GLP1  - Mounjaro  - Semaglutide  - Weight loss drug |
| **Related to fertility** | 70 | 88 |  |
| **Positive** | 37 (51.85%) | 48 (54.54%) |  |
| **Neutral** | 21 (30%) | 29 (32.95%) |  |
| **Negative** | 12 (17.14%) | 11 (12.5%) |  |
| **Notes:** | - improved PCOS symptoms  - be careful with GLP1 + pregnancy  - lack of research on pregnancy and after stopping  - more personal anecdotes | - more news tweets, so neutral stance  - skepticism about enough research “too good to be true”  - PCOS awareness  - more stances on how society will change if ozempic is marketed as a fertility drug (infertility crisis in the US)  - issues about price + accessibility  - few tweets about male infertility too  - “Cautious optimism” |  |

| **Pubmed search for GLP-1 agonist with:** | **Peer-reviewed studies** |
| --- | --- |
| **Women with PCOS** | 112 total:  52 original articles  60 review article |
| **Women without PCOS** | 0 |
| **Ovarian reserve** | 1 in animal model |
| **Oocyte physiology** | 1 in animal model |
| **Menstrual irregularities** | 1 review article |
| **IVF** | 1 original article in PCOS |
| **Granulosa cell physiology** | 3 in animal model |
| **Endometriosis** | 0 |
